# Canonical NFκB signaling in myeloid cells is required for the glioblastoma growth

**DOI:** 10.1038/s41598-017-14079-4

**Published:** 2017-10-23

**Authors:** B. R. Achyut, Kartik Angara, Meenu Jain, Thaiz F. Borin, Mohammad H. Rashid, A. S. M. Iskander, Roxan Ara, Ravindra Kolhe, Shelby Howard, Natasha Venugopal, Paulo C. Rodriguez, Jennifer W. Bradford, Ali S. Arbab

**Affiliations:** 1 Tumor Angiogenesis Laboratory, Biochemistry and Molecular Biology, Georgia Cancer Center, Augusta University, Augusta, GA USA; 2 Department of Pathology, Georgia Cancer Center, Augusta University, Augusta, GA USA; 3 Cancer Immunology, Inflammation and Tolerance Program, Georgia Cancer Center, Augusta University, Augusta, GA USA; 4 Department of Biological Sciences, Augusta University, Augusta, GA USA

## Abstract

Tumor development and therapeutic resistance are linked with tumor-associated macrophage (TAM) and myeloid-derived suppressor cell (MDSC) infiltration in tumors via chemokine axis. Chemokine expression, which determines the pro or anti-inflammatory status of myeloid cells, are partly regulated by the nuclear factor-kappa B (NF-κB) pathway. Here, we identified that conditional deletion of canonical NF-κB signaling (p65) in myeloid cells inhibited syngeneic glioblastoma (GBM) through decreased CD45 infiltration in tumors, as characterized by decreased TAMs (CD206+) and MDSCs (Gr1+ CD11b+), increased dendritic cells (CD86+) and cytotoxic T cells (CD8+) in the p65 knockout (KO) mice. Proinflammatory cytokines (IFNγ, MCP1, MIP1α, and TNFα) and myeloid differentiation factor (Endoglin) were increased in myeloid cells from p65 KO tumor, which demonstrated an influence on CD8+T cell proliferation. In contrast, p65KO athymic chimeric mice with human GBM, failed to inhibit tumor growth, confirming the contribution of T cells in an immune competent model. The analysis of human datasets and GBM tumors revealed higher expression of p65 in GBM-associated CD68+ macrophages compared to neighboring stroma. Thus, canonical NF-κB signaling has an anti-inflammatory role and is required for macrophage polarization, immune suppression, and GBM growth. Combining an NF-κB inhibitor with standard therapy could improve antitumor immunity in GBM.

## Introduction

Glioblastoma (GBM), a grade IV astrocytoma as classified by World Health Organization, is a highly malignant, vascular, and invasive subtype^1^. Hypoxia and neovascularization are signature histopathologic features of GBM^2^, which is most lethal during the first year after initial diagnosis, despite surgical resection and other standard therapies^1,3^. Recent reports suggest that tumor growth depends on the tumor microenvironment (TME)^4^. Peripheral macrophages and microglia are the most abundant non-cancerous cell types in GBM, in some cases accounting for up to 30% of the total tumor composition^5,6^. Tumor-associated hypoxia is known to upregulate hypoxia inducible factor 1-α (HIF1-α), transcribe stromal cell-derived factor 1α (SDF-1α), and promote secretion of proangiogenic factors to recruit CXCR4+ bone marrow-derived cells (BMDCs) in the tumor milieu^7–10^. The myeloid populations of BMDCs, such as tumor-associated macrophages (TAMs) and immune regulatory myeloid-derived suppressor cells (MDSCs), are critical in tumor development^11,12^. TAMs in the TME are skewed towards an M2 polarized state and are a central target in cancer therapy^13^. Several chemokines, such as macrophage colony stimulating factor-1 (m-CSF/CSF1) and monocyte chemotactic protein-1 (MCP1/CCL2) are known to contribute to the recruitment of heterogeneous myeloid cells to the tumors due to the presence of CSF1 receptor (CSF1R)^14–16^.

Chemokines and pro-inflammatory peptides are often expressed in response to the induction of expression of nuclear factor-κB (NF-κB) by cytokines or other stimuli in cancer^17,18^. Chemokines are critical in regulating cancer-associated transport, activation, and proliferation of several cell types, including myeloid, lymphoid, endothelial and epithelial cells^19,20^. Previously, we identified that chemokine signaling, especially through CXCL7, plays a key role in GBM growth and antiangiogenic therapy resistance. Targeting CSF1R+ myeloid cells significantly decreased CXCL7 and thus the GBM growth^12^. Interestingly, chemokines, including CXCL7, are secreted by the host peripheral macrophages and are regulated through the NF-κB signaling in murine models^17^. In human TAMs, CXCL8 or IL8 expression is mediated through NF-κB driven transcription in response to m-CSF and MCP1^21^. Moreover, it has been widely recognized that chemokines are one of the major targets of canonical NF-κB signaling.

NF-κB is considered as a master regulator of inflammation mechanisms, is increasingly recognized as a crucial player in many steps of cancer initiation and progression, and thus serves as a critical link between inflammation and cancer^22^. NF-κB follows p50 and p65 (RelA) mediated canonical as well as p52 and RelB mediated non-canonical pathways^23–25^. NF-κB cross-talks with different kinases, such as GSK3-β, p38, or PI3K, which modulate the NF-κB transcriptional activity or affect upstream signaling pathways^26^. NF-κB cooperates with multiple transcription factors in pathways such as STAT3 and p53, which either directly interact with NF-κB subunits or affects NF-κB target genes in the nucleus. Depending on the context, such as in different tumor types, NF-κB signaling could be tumor promoting or anti-tumorigenic in cancer cells and their microenvironment^27^. It has recently been shown that NF-κB signaling can drive GBM cancer stem cells^28^, but surprisingly, no data is available in the GBM microenvironment, and it is not understood whether the canonical NF-κB pathway has a proinflammatory or anti-inflammatory role in GBM tumor recruited myeloid cell populations.

The present study is focused on studying myeloid cell-associated canonical NF-κB signaling with a special interest in GBM models. We identified that deleting myeloid cell associated NF-κB signaling resulted in M2 to M1 polarization and enhancement of CD8+T cell-mediated antitumor immunity in an immune competent mouse model. Further, data were validated in an immunocompromised athymic nude chimera model, which showed tumor growth advantages in the absence of a T cell component. Here, we report for the very first time that GBM growth is influenced by myeloid cell-associated NF-κB signaling in the host through anti-inflammatory phenotypes, and is a potential target for GBM therapy. We anticipate that combining standard temozolomide therapy with a pharmacological NF-κB inhibitor could improve the outcome of GBM treatments in the clinic.

## Results

### Myeloid-specific deletion of NF-κB signaling favors syngeneic GBM growth inhibition in an immune competent host

**Figure 1:**
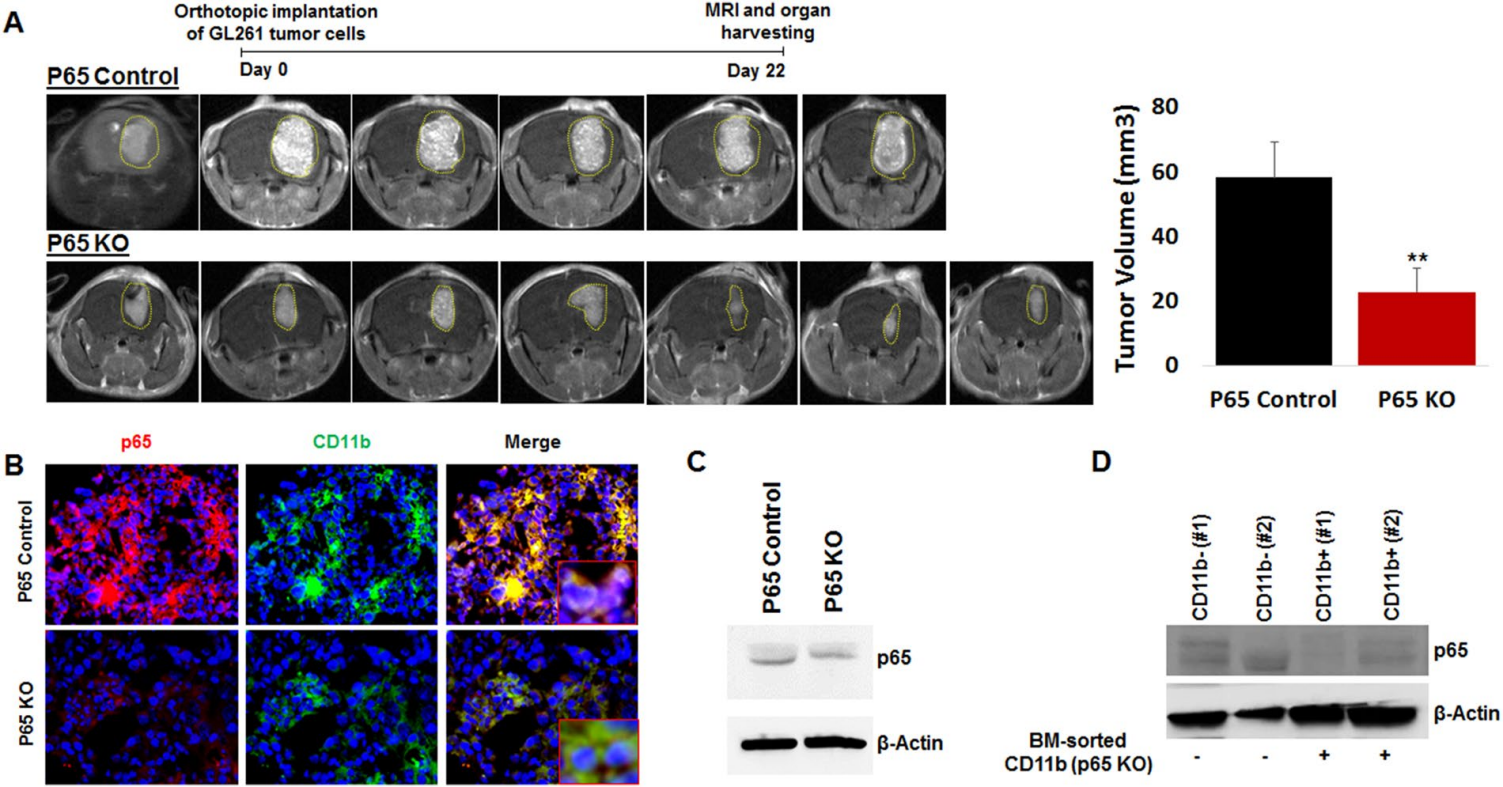
Myeloid-specific deletion of NF-κB signaling decreases syngeneic GBM growth. (**A**) Syngeneic GL261 GBM tumors were developed in p65 control and p65 KO mice. MRI data showing significantly decreased GBM tumors in p65KO mice compared to controls. (**B**) Tissue-based immunofluorescence images showing decreased p65 expression in myeloid cells from p65 KO tumors compared with control tumors. (**C**) Brain sorted microglia displayed comparable p65 expression in both control and p65 KO mice. (**D**) Western blot data showing p65 expression in bone marrow sorted CD11b+ and CD11b− cells. The p65 KO mice, which were identified for incomplete p65 loss at the protein level in CD11b+ cells (e.g. mouse #2), were excluded from the study. Shown is one of the two experiments performed. Quantitative data is expressed in mean ± SD. **P < 0.01.

Canonical NF-κB signaling in the myeloid cell lineage was inhibited through the LysM-Cre-mediated conditional deletion of the p65 subunit. Lysozyme (LysM) Cre (B6.129P2-*Lyz2* ^*tm1(cre)Ifo*^/J) male mice were crossed with p65^flox/flox^ female mice on the C57BL6 background, resulting in conditional p65 deletion in cells of the myeloid compartment. p65^fl/fl^/LysMCre (KO), (n = 7) and LysMCre control (n = 6) mice were used to develop syngeneic GL261 GBM tumors following orthotopic tumor cell implantation at day 1, as shown previously by our laboratory^12,29^. Magnetic resonance imaging (MRI) was used to monitor tumor growth on day 22 following implantations (end-point). MRI data analysis identified significantly decreased GBM tumor growth in p65KO mice compared to controls (Fig. 1A, left and right panels). The MRI data corroborated with the histology data (Supplementary Figure 1) showing decreased tumor burden. Interestingly, tumors implanted in p65KO mice showed decreased overall proliferation (Ki67 expression) and increased overall apoptosis (cleaved caspase 3 expression), as compared to p65 control tumors (Supplementary Figure 2A and B). GL261 GBM tumor bearing p65KO and control mice were analyzed for p65 loss in the myeloid compartment using tissue-based immunofluorescence, which identified decreased CD11b+ cells as well as decreased p65 protein expression in CD11b+ cells in p65KO compared to control mice (Fig. 1B). Interestingly, CD11b+ cells in control mice depicted high p65 expression, probably due to the tumor-induced pathological hypoxic environment (Figs 1B and 6C). Interestingly, brain microglia displayed a comparable level of p65 expression in p65 KO mice as compared to controls (Fig. 1C and Supplementary Figure 3 left panel). Furthermore, bone marrow sorted CD11b+ and CD11b- cells were probed for p65 loss of expression. Although LysMcre performs incomplete excision in some mice (50–70%)^30^, it has been a widely used model in delineating myeloid cell function^31,32^. Any p65 KO mice, which were identified for incomplete p65 loss at the protein level in CD11b+ cells (e.g. lane 4 in Fig. 1D and Supplementary Figure 3 right panel), were excluded from the study.

### Myeloid-specific deletion of NF-κB signaling polarizes cell microenvironment in an immune competent host

**Figure 2:**
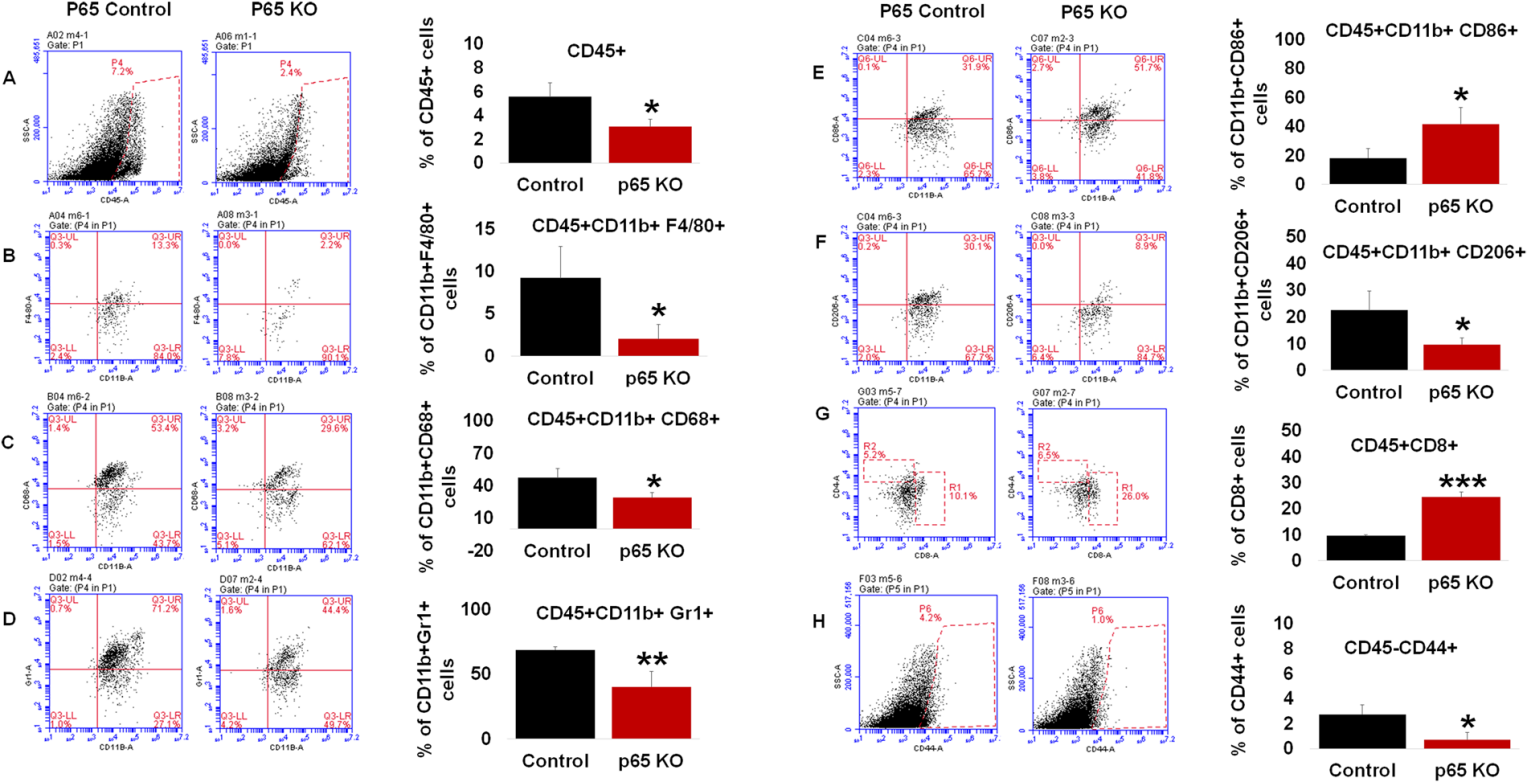
Myeloid-specific deletion of NF-κB signaling polarizes microenvironment: Flow cytometry data showing (**A**) Myeloid-specific deletion of NF-κB signaling resulted in decreased infiltration of bone marrow-derived CD45+ leukocytes in the TME compared with the control mice. (**B**) CD45+ leukocytes were characterized as decreased common macrophages (F4/80+), (**C**) tumor associated macrophages (CD68+), (**D**) decreased MDSCs (Gr1+ CD11b+), (**E**) increased M1 macrophages (CD86+), (**F**) decreased M2 macrophages (CD206+ or mannose receptor), (**G**) increased CD8+T cells in the p65 KO tumors compared to control tumors. (**H**) Flow cytometry data showing decreased CD45− and CD44+, mesenchymal stem cell (MSCs)-like cells in the p65 KO tumors compared to control tumors. Shown is one of the two experiments performed. Quantitative data is expressed in mean ± SD. *P < 0.05, **P < 0.01 and ***P < 0.001.

GL261 GBM tumor bearing p65KO (n = 3) and control mice (n = 3) were analyzed for cellular differences in the tumor microenvironment. p65KO mice implanted with GBM had statistically significant cellular reductions in CD45+ leukocyte (Fig. 2A), macrophage (F4/80+) (Fig. 2B), tumor-associated macrophage (CD68+) (Fig. 2C), MDSC (Gr1+ CD11b+) (Fig. 2D), and M2 macrophage (CD206+ or mannose receptor) (Fig. 2F) populations. Increases in cellular populations were seen in M1 macrophages (CD86+) (Fig. 2E) and CD8+T cells (Fig. 2G), with an increasing trend seen in CD4+T cells (Supplementary Figure 4A) in the p65 KO tumors compared to control tumors. Interestingly, CD45−/CD44+ tumor cells (a mesenchymal marker) were decreased (Fig. 2H), but not CD45-/CD133+ tumor cells (hematopoietic marker) (Supplementary Figure 4B) in the p65 KO tumors compared to control tumors. We tested the immunogenicity potential of GL261 that express about 48% MHC1 (Supplementary Figure 3C). This data along with other data in the manuscript indicate that myeloid cell-associated and p65 mediated NFkB signaling is critical in immunogenic tumors. We suspected that there was myeloid NF-κB signaling-mediated regulation of mesenchymal stem cells (MSCs)^33^, therefore, we performed tumor cell (GL261 and HF2303) spheroid formation assays. No difference in spheroid size was observed in a cell culture experiment in the presence of 50% conditioned medium derived from 24 hours’ culture of wild-type, control or p65 KO BMDMs. (Supplementary data 5A and B). This indicated no significant contribution of myeloid cell-mediated NF-κB signaling in GBM stemness.

### Myeloid-specific deletion of NF-κB signaling resulted in an anti-tumor secretome in an immune competent host

**Figure 3:**
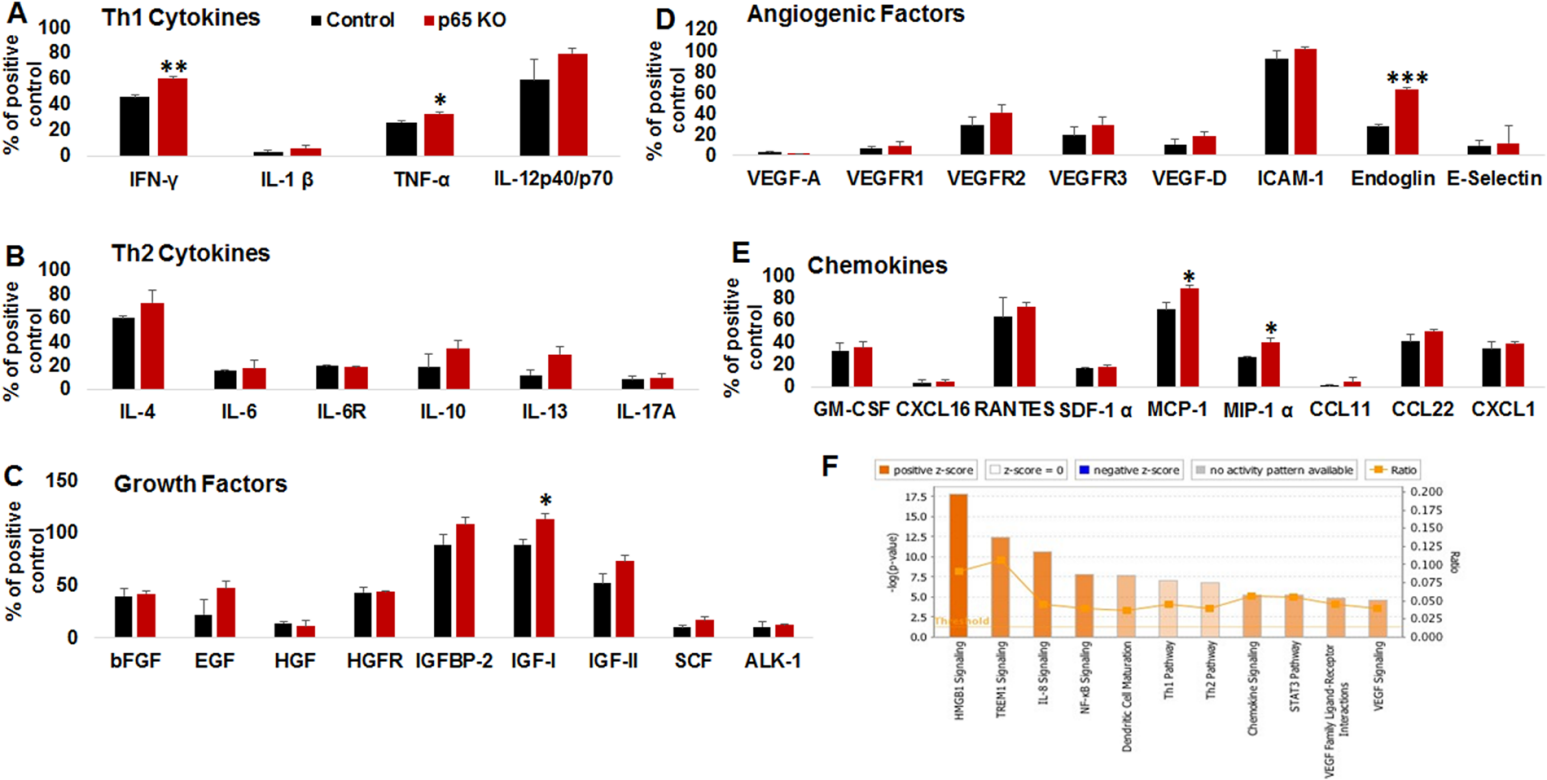
Myeloid-specific deletion of NF-κB signaling displayed anti-tumor secretome: Membrane-based cytokine array data showing (**A**) increased expression of IFN-γ and TNF-α (Th1 cytokine), (**B**) no change in Th2 cytokines, (**C**) increased expression of IGF-1 (growth factor), (**D**) increased expression of endoglin (myeloid maturation factor), and (**E**) increased expression of MCP-1 and MIP-1α (chemokines) in p65 deleted CD11b+ myeloid cells compared to p65 control myeloid cells. (**F**) web-based pathway analysis discovered that significantly altered factors predominantly belonged to the HMGB1 signaling pathways. Interestingly, altered factors also found to be associated with top 4 pathways TREM1 > IL8 > NF-κB > dendritic cell maturation. Shown is one of the two experiments performed. Quantitative data is expressed in mean ± SD. *P < 0.05, **P < 0.01 and ***P < 0.001.

At the day 22 end-point (see Fig. 1A), GL261 tumors were isolated from p65 control (n = 3) and p65 KO mice (n = 3) following MRI. Freshly **s**orted CD11b+ cells from the GL261 TME were subjected for protein lysate preparation followed by cytokine protein arrays. The customized array involved 44 factors with Th1 and Th2 cytokines, growth factors, angiogenic factors, and chemokines (Fig. 3A–E). The analysis identified increased levels of IFN-γ and TNF-α (Th1 cytokines), IGF-1 (growth factor), endoglin (differentiation and phagocytosis), and MCP-1 and MIP-1α (chemokines) in p65 deleted myeloid cells compared to p65 control myeloid cells. No change in Th2 cytokines was seen in comparison. Further, web-based pathway analysis discovered that the significantly altered factors predominantly belonged to the HMGB1 (proinflammatory pathway) and TREM1 signaling networks. IL8, NF-κB, and dendritic cell maturation pathways were also implicated, as shown in Fig. 3F and Supplementary Figure 6A–C.

### Myeloid-specific deletion of NF-κB signaling induces T cell proliferation, CD8+T cell polarization, and Th1 cytokine production in coculture

**Figure 4:**
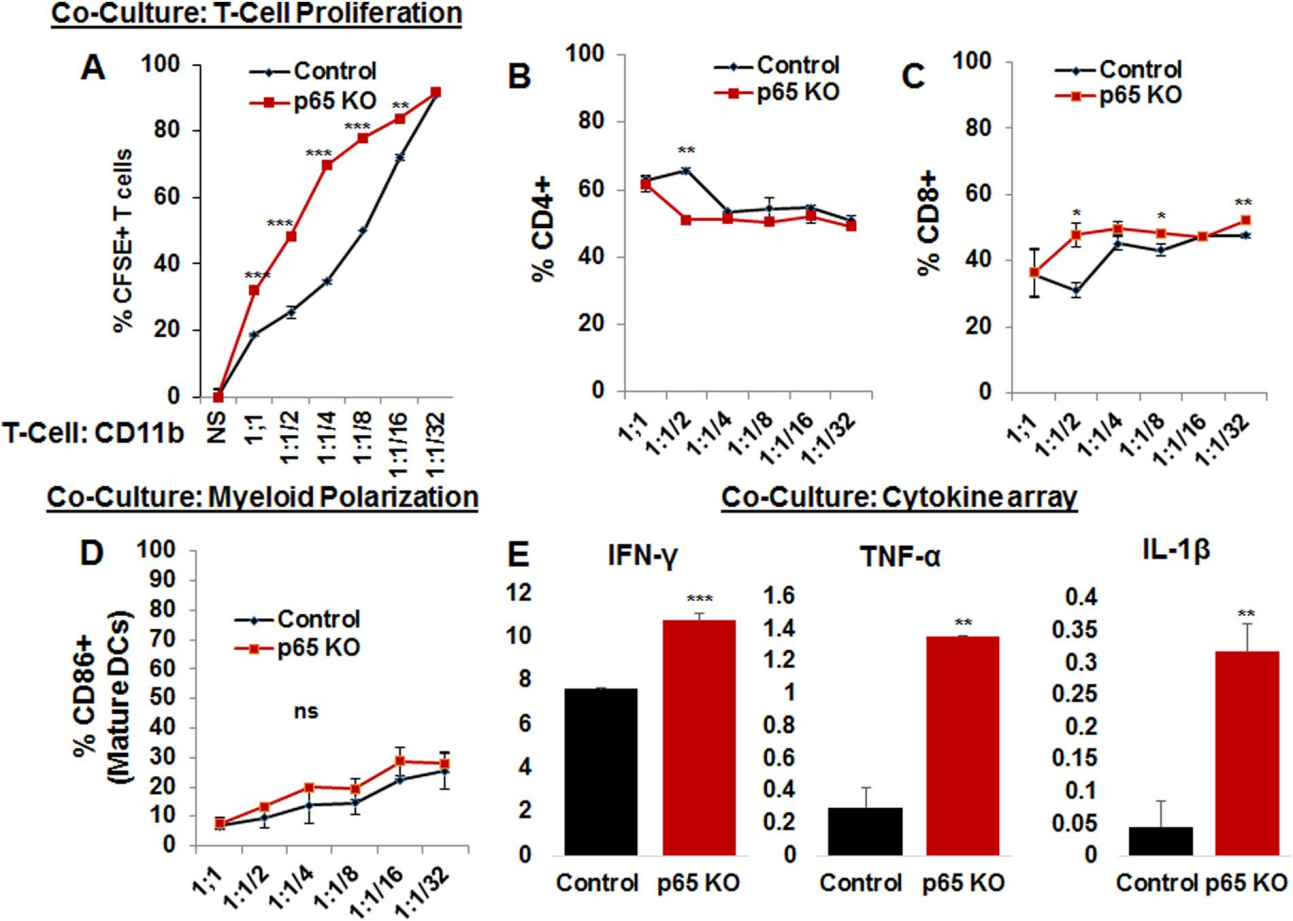
Myeloid-specific NF-κB signaling inhibits T cell proliferation: *In vitro* data showing co-culture experiments with sorted and CFSE labeled CD3+T cells and tumor isolated CD11b+ from p65KO or p65 control mice. (**A**) flow cytometry data showing that tumor sorted CD11b+ cells from p65 KO mice displayed higher T cell proliferation at 1:1, 1:1/2, 1:1/4, 1:1/8 and 1:1/16 compared to control CD11b+ cells. (**B**) CD4 proliferation was decreased at the 1:1/2 co-culture and (**C**) CD8 proliferation was increased at the 1:1/2, 1:1/8 and 1:1/32 co-cultures in the p65KO group compared to p65 control group. (**D**) flow data showing an increasing trend of CD86+ mature DCs in p65KO co-cultures compared to p65 controls. (**E**) analysis of co-culture supernatant using membrane-based cytokine array depicted higher levels of IFN-γ, TNF-α, and IL1-β (Th1 cytokines) in p65KO co-culture (1:1) compared to p65 controls. Shown is one of the two experiments performed. Quantitative data is expressed in mean ± SD. *P < 0.05, **P < 0.01 and ***P < 0.001.

Increased CD8+T cells in p65KO compared to p65 control group suggested a drastic effect of myeloid NF-κB signaling on T cells. Therefore, coculture experiments were performed where normal spleen and lymph node sorted CD3+T cells were labeled with CFSE and then cocultured with tumor associated CD11b+ cells isolated from p65KO (n = 4) or p65 control (n = 4) mice. Different ratios of T cell: CD11b cell cocultures were performed, ranging from 1:1 to 1:1/32 (Fig. 4A). Interestingly, tumor sorted CD11b+ cells from p65 KO mice drove higher T cell proliferation at 1:1, 1:1/2, 1:1/4, 1:1/8 and 1:1/16 as compared to tumor sorted CD11b+ cells from p65 controls (Fig. 4A). Surprisingly, CD4 proliferation was decreased at the 1:1/2 co-culture ratio (Fig. 4B and Supplementary Figure 7A) and CD8 proliferation was increased at the 1:1/2, 1:1/8 and 1:1/32 co-culture ratios in the p65KO group compared to p65 control group, indicating an antiproliferative role of myeloid canonical NF-κB signaling on CD8+T cells under tumor condition (Fig. 4C and Supplementary Figure 7B). There was an increasing trend of mature DCs in p65KO co-cultures compared to p65 control co-cultures, suggesting autocrine, as well as paracrine, effects in coculture (Fig. 4D). In addition, analysis of coculture supernatant showed increased levels of IFN-γ, TNF-α, and IL1-β (Th1 cytokines) in p65KO co-culture compared to p65 control co-culture (1:1) (Fig. 4E). We noticed the similar effect on CD4 and CD8 T cell proliferation when stimulated T cells were treated with IFN-γ (100 U/ml), TNF-α (100 ng/ml), or IL1-β (100 ng/ml), or a combination of the three in 72 hours of culture study (Supplementary Figure 8).

### Bone marrow chimera with myeloid-specific deletion of NF-κB signaling failed to inhibit human GBM growth in an immune-deficient host

**Figure 5:**
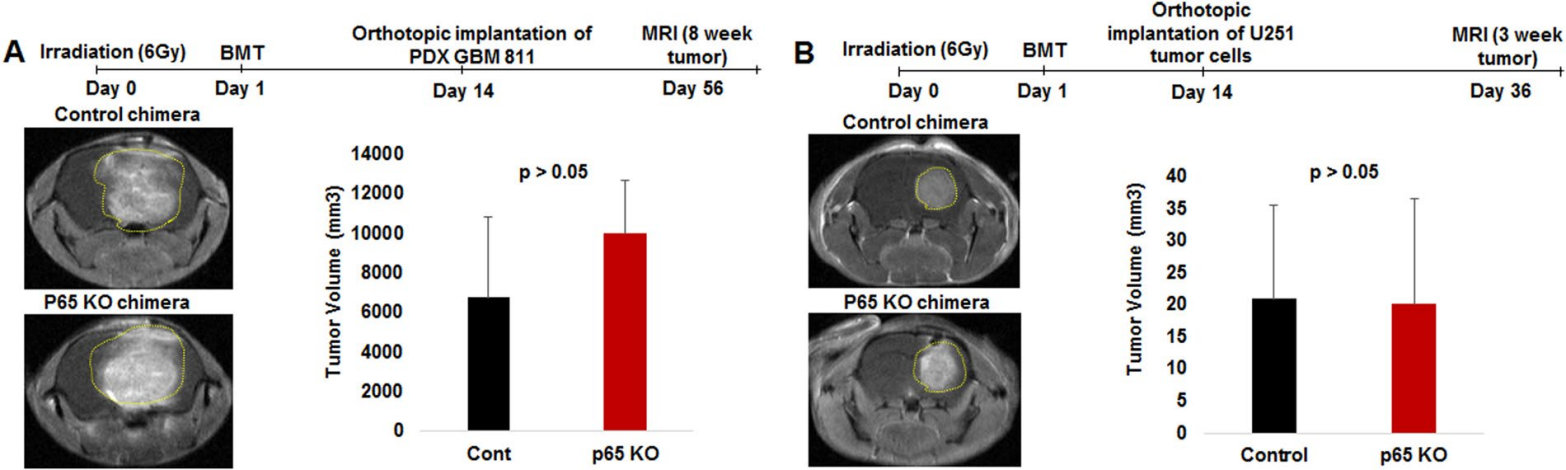
p65 KO chimera failed to inhibit human GBM growth in immune-deficient host. (**A** and **B**) The p65 KO and control recipient mice bone marrow was isolated and transplanted into irradiated donor nude mice. Once mice achieved 70% engraftment (chimera) in 2 weeks, U251 or PDX GBM811 cells were orthotopically implanted and followed-up for MRI at the 3-week or 8-week protocol, respectively. MRI data showing p65KO chimera failed to decrease the U251 or PDX GBM811 human tumor growth. Shown is one of the two experiments performed. Quantitative data is expressed in mean ± SD.

The syngeneic tumor growth patterns were validated using human tumors in immune-deficient mice. p65 KO and control donor mouse bone marrow was isolated and transplanted into irradiated recipient athymic nude mice (Fig. 5A and B). As shown in our previous study, more than 70% bone marrow reconstitution or engraftment in irradiated mice takes place 2 weeks following intravenous injection of 5–10 × 10^6^ cells^12,34^. Once mice achieved 70% engraftment (chimera), U251 (n = 6) or PDX GBM811 cells (n = 6) were orthotopically implanted and followed-up by MRI at the 3-week or 8-week protocol, respectively (Fig. 5A and B)^12,29^. Surprisingly, p65KO chimera in athymic nude mice did not decrease the U251 or PDX GBM811 human tumor growth, suggesting a critical role of T cells in anti-tumor immunity. In fact, PDX GBM811 tumor growth was even slightly increased as compared to control chimera (Fig. 5A and B). Analysis of human TME (GBM811) identified that p65KO chimera had increased total CD45+ leukocytes, total CD11b+ cells, F4/80+ macrophages, CD68+ macrophages, Gr1+ CD11b+ (total MDSCs), Ly6G+ CD11b+ (granulocytic MDSCs), CD11b+ CD86+ (mature DCs or M1 macrophages), and CD11b+ CD206+ (M2 macrophages), but decreased Ly6C+ CD11b+ (monocytic MDSCs) as compared to TME in control chimera (data not shown; Supplementary data 9A–F). Human cancer stem cells (CSCs: CD45-CD49f+, CD45-CD15+ or CD45-CD44+) were comparable in p65KO and control TME in chimera model (data not shown).

### Canonical NF-κB signaling is associated with human brain tumors

**Figure 6:**
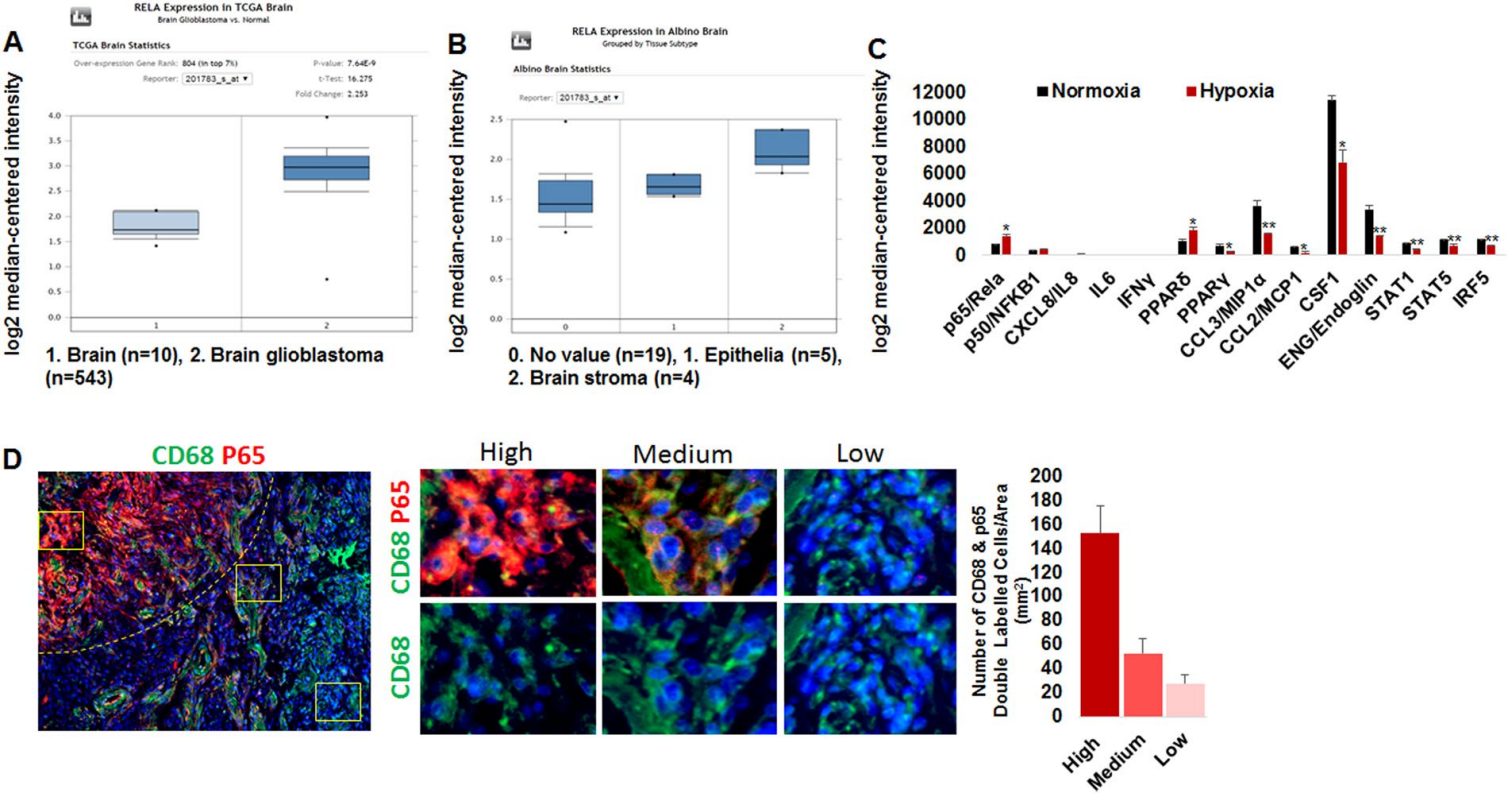
Elevated stromal NF-κB signaling in human brain tumors. (**A**) The TCGA brain data showing increased p65 expression in GBM compared to normal brain. (**B**) Analysis of tumor and stroma in Albino brain dataset identified increased p65 expression in stroma compared to tumor compartment. (**C**) GEO dataset (GSE4630) showing hypoxia-induced p65 mRNA expression associated mediators involved in inflammation and myeloid differentiation in hypoxic human macrophages compared to macrophages cultured in normoxia. (**D**) Analysis of paraffin fixed GBM brain tissues identified heterogeneous p65 protein expression pattern in CD68+ myeloid cells.

In order to put our data into context with human disease, we first analyzed mRNA expression data from publicly available brain cancer patient datasets. The Cancer Genome Atlas (TCGA) brain data, with 10 normal adjacent and 542 GBM brain samples, suggested there is significantly increased RELA (p65) expression (canonical NF-κB signaling) in GBM (2.25 fold) as compared to normal brain (Fig. 6A). Analysis of brain tumor (n = 5) and stroma (n = 4) identified increased RELA expression in stroma as compared to tumor compartments in the Albino brain oncomine dataset (Fig. 6B). Pathological p65 expression in stroma or tumor associated myeloid cells was further analyzed through the GEO dataset (GSE4630)^35^. The study utilized buffy coat isolated human mononuclear cells followed by culture under hypoxia (1% O_2_) and normoxia (21% O_2_) conditions. We identified that hypoxia-induced p65 mRNA expression in human macrophages compared to macrophages cultured in normoxia. The p65 overexpression under hypoxia was associated with decreased pro-inflammatory and cell differentiation mediators such as PPARγ, MIP1α, MCP1, CSF1, Endoglin, STAT1, STAT5, and IRF5 (Fig. 6C). This was further confirmed by the analysis of paraffin fixed GBM patient brain tissues (n = 4), which identified a heterogeneous p65 protein expression pattern in CD68+ myeloid cells (Fig. 6D). High p65 expression in CD68+ macrophages was seen at the inner invasive compartment of the tumor-associated stroma. Medium p65 expression was noticed at the outer invasive compartment of the tumor. Correspondingly, medium p65 expressive CD68+ myeloid cells were found around the blood vessel areas. In addition, low or non-p65 expressive CD68+ myeloid cells were found mostly away from the invasive and hyper vascular tumor compartments (Fig. 6D, right panels).

## Discussion

Conflicting data exists in the literature, with the majority from the immunology field, suggesting the production of pro-inflammatory or immune suppressive mediators in the presence of active canonical NF-κB signaling in myeloid cells. NF-κB signaling has been known to regulate macrophage-driven inflammation in hematological and autoimmune diseases^18,36–38^. NF-κB pathway through Notch 1 signaling activates microglia following hypoxia exposure^39^. The canonical NF-κB pathway is required for the human myeloid dendritic cell development and function^40^, however, NF-κB signaling also regulates M2 polarization^41^. Recently, NF-κB signaling was found to be a mediator of MDSC expansion in an aging model^42^. In addition, noncanonical NF-κB signaling was reported to mediate STAT3-stimulated IDO upregulation, an immune suppressive mediator in MDSCs^43^. The present study identified that tumor-associated myeloid cells deficient in NF-κB signaling display anti-inflammatory properties, which was evident by increased inflammatory mediators following conditional deletion of p65 in myeloid cells in our GBM model.

First, we observed that p65 KO mice had reduced syngeneic GBM growth. Thus, canonical NF-κB signaling in myeloid cells is required for the GBM growth. Our tumor growth data corroborates with previous reports: In a colitis-associated cancer model, deletion of a canonical NF-κB inhibitor of nuclear factor kappa-B kinase (IKK2) in the myeloid lineage led to a reduction in tumor incidence and size^44^. In an ovarian tumor model, transplantation of macrophages with inhibited IKK2 led to a reduction in tumor burden through the switch from M2 to M1 macrophage phenotype^45^. In a hepatocellular carcinoma model, targeting an NF-κB signaling mediator, an IκB-super repressor, in the liver macrophages (Kupffer cells) led to a decreased tumor incidence^46^. NF-κB signaling, particularly the p65 subunit, has been shown to modulate M2 polarization in a hepatoma model, where tumor cells produced TLR2-related ligands, which are capable of activating p65 nuclear translocation in TAMs^47^. After turning on related gene expression, nuclear p65 is exported into the cytoplasm for autophagosomal degradation which limits NFκB activity and drives TAM to M2 macrophage polarization^47^. In the present study, we anticipated that deletion of NF-κB signaling in myeloid cells may limit the above mechanisms of M2 polarization, which was evident by the decreased M2 (CD206) macrophages and increased M1 (CD86) macrophages in GBM tumor-bearing p65 KO mice compared to control mice. Interestingly, NOS2+ macrophages (M1) through NO production were found to be involved in T cell infiltration and tumor rejection^48^. Similarly, we also noticed that increased M2 to M1 polarization and CD8+ infiltration in tumor-bearing p65 KO mice as compared to control mice. Interestingly, one study has identified the anti-tumor role of macrophage-associated NF-κB signaling in mammary tumor-mediated lung metastasis model^49^. Moreover, most studies suggest a tumor-promoting effect of NF-κB in myeloid cells, including TAMs. Despite these data pointing to a pro-tumor role for NF-κB in macrophages, genes regulated by NF-κB could also lead to an anti-tumor phenotype, suggesting that effects may be more complex. Altogether, the extent of NF-κB signaling driven mechanisms in critical immune cells such as cancer-associated heterogeneous myeloid cell populations are poorly understood and controversial in solid cancers^49,50^.

NFκB is a key transcription factor in controlling gene expression during monocyte/ macrophage activation^51^. The macrophage-derived cytokines IL-1β and TNF-α, are potent activators of NF-κB. In turn, their expression is controlled by NF-κB, thus creating a positive feedback loop. Interestingly, physical and oxidative stress mediators, such as NOS2 or COX-2, may induce NFκB pathway in myeloid cells^52,53^. We found that p65 deleted myeloid cells produced an increased amount of IFN-γ, TNF-α, IGF1, endoglin, MCP-1 (CCL2) and MIP-1α (CCL3). Interestingly, these factors belong to HMGB1 signaling pathways identified in a web-based pathway analysis. HMGB1 has been shown to activate pro-inflammatory signals in the literature^54–56^ and promote autophagy of tumor-associated myeloid cells such as MDSCs^57^. This could explain the decreased MDSCs and pro-tumor TAMs in p65KO group compared to p65 controls. Further, T cell proliferation, especially CD8+T cells, was increased following T cell coculture with p65KO myeloid cells compared to control tumor myeloid cells. Co-culture supernatant with p65KO myeloid cells was characterized to have increased IFN-γ, TNF-α and IL-1β compared to control myeloid cells. It is anticipated that IFN-γ, TNF-α and IL-1β cytokines were produced by the p65 deleted myeloid cells in the coculture, which supported the T cell survival and proliferation. The culture of T cells with IFN-γ, TNF-α, and IL1-β cytokine showed similar T cell proliferation patterns. These data indicate that proinflammatory cytokines IFN-γ, TNF-α, and IL1-β have a proliferative effect specifically on the CD8+T cell population and an antiproliferative effect on the CD4+T cell population. This study result corroborates with our previous co-culture study findings in manuscript (Fig. 4B and C). Previously published studies also justify our observation, where authors found a direct effect of IFN-γ on T cells in the regulation of CD8 T cell homeostasis^58^. In addition, it is anticipated that compensation or alternative mechanisms play a critical role in producing proinflammatory cytokines in p65 deleted tumor-associated myeloid cells in the presence of host T cells. These alternative mechanisms must be investigated more thoroughly to understand the NF-κB signaling independent regulation of pro-inflammatory cytokine production under intricate tumor conditions^26^.

Previous reports indicated the involvement of NF-κB in T-cell proliferation and activation^59^. Our data support an anti-inflammatory role of myeloid NF-κB signaling in GBM, which was evident by the production of proinflammatory cytokines by p65 deleted myeloid cells in TME as well as in coculture with the T cells. Mouse data was corroborated with the human GBM datasets where the p65 subunit was overexpressed in the tumor-associated stroma (Fig. 6). Further, a human macrophage culture data set identified overexpression of p65 following hypoxia which was associated with the decreased pro-inflammatory and cell differentiation mediators, such as PPAR-γ, MIP1-α, MCP1, CSF1, Endoglin, STAT1, STAT5 and IRF5 (Fig. 6)^60,61^. Interestingly, decreased Endoglin expression is associated with the immature status of myeloid cells, causing impaired immune responses^62^. The anti-inflammatory role of NF-κB signaling has been reported earlier^63^. The previous study noticed that p50 (NFKB1) overexpression in canonical NF-κB signaling inhibited proinflammatory IL-12 cytokine expression in normal macrophages. TAMs isolated from p50 knockout mice showed normal production of M1 cytokines and were associated with the decreased tumor growth. Thus, p50 overexpression accounts for the inability of TAMs to mount an effective M1 antitumor response capable of inhibiting tumor growth^63^. Nevertheless, these data indicate that canonical NF-κB signaling has anti-inflammatory effects, which favor GBM tumor growth. However, the expression of proinflammatory cytokines in p65 deleted myeloid cells is extremely complex and requires future investigation in the context of T cell function. Moreover, studies support that NF-κB signaling through context-dependent functions may play a pivotal role in governing heterogeneous myeloid cell function during inflammation and cancer development. Please note that microenvironment with intact immune system play a critical role here because anti-tumor effects were only seen in syngeneic tumor model (GL261) in immunocompetent animals. Other human tumor models in immune compromised mice with p65^flox/flox^ control or p65 KO chimera did not decrease in tumor growth. We anticipate that p50 dimers might have inhibited the expression proinflammatory cytokines (such as TNFα, IFNγ, and IL1β) through inhibiting the p50-p65 complex in the nucleus in control mice. However, p50 dimers would not be able to suppress the proinflammatory cytokine promoter due to absence of p65 (p50-p65 complex) in myeloid cells in p65 KO mice^63–65^. Please see Fig. 3 indicating the increase in pro-inflammatory cytokines in p65 KO mice compared to control mice. Nevertheless, this human data fully validates our findings in Fig. 3 that why p65 deleted myeloid cells have increased expression of selective proinflammatory cytokines/chemokines.

Previously, it was identified that macrophages induce invasiveness of cancer cells and CSCs in breast cancer model^33^. Our *in vitro* data showed no change in invasive potential or sphere-forming potential following incubation of conditioned medium of normal BMDM, p65 KO or control BM cells cocultured with GL261 or HF2303 tumor cells. Therefore, the decrease in CD44+ CSCs in invasive GL261 tumors in p65 KO mice compared to controls could be the result of the anti-tumor effect of CD8+T cells in syngeneic GBM model. This is further evidenced by the human PDX GBM 811 or U251 tumors in chimeric nude models, which showed comparable CD44+ CSCs in TME in the absence of host T cell component. Moreover, the present study supports that CSC phenotypes in the immunocompetent hosts are mediated through the NFκB signaling in macrophages and cytotoxic CD8+T cells. However, a functional stemness assay such as limiting dilution transplantation between GBM tumors in control and p65KO mice, which is out of the scope of the present study, may provide a better understanding of the role of myeloid NFkB in regulating stemness in GBM models.

We investigated that myeloid cell-associated and p65 mediated NFkB signaling is critical in immunogenic tumors such as a murine GL261 tumor. Please note that human tumors are higher in immunogenicity, for example, a study by Jacobs *et al*., reported that human cell line models such as U251 and U87 are more immunogenic compared to GL261 model^66^. Therefore, using a low immunogenic cell line may not reflect the clinical scenario in our study. Unfortunately, GL261 is the only plausible syngeneic murine cell line available, as it recapitulates human glioblastoma characteristics such as hypervascularity, hypoxia, and hyper-invasive phenotypes^67^. GL261 also depicts a mutation status similar to human tumors and is very well characterized for cancer therapeutic studies^66–68^.

**Figure 7:**
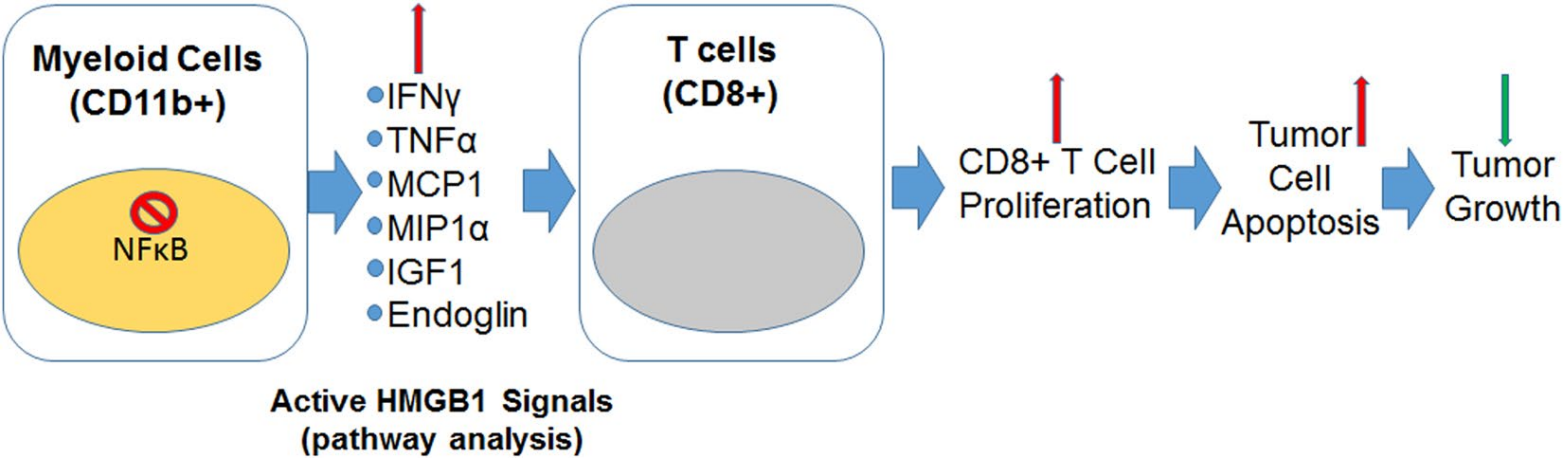
Schematic showing increased expression of pro-inflammatory HMGB1 signals in canonical NF-κB deleted myeloid cells modulate proliferation of CD8+T cells and increase apoptosis in the TME to limit GBM growth.

In summary, we identified that myeloid NFκB signaling is heterogeneous in the human GBM-associated stroma. The present study identified the anti-inflammatory role of myeloid NFκB signaling in promoting GBM tumor growth through immune suppression mechanisms. Therefore, targeting/inhibiting myeloid-specific NFκB signaling in GBM could inhibit the immune suppressive TAMs and improve the anti-tumor immunity (Fig. 7). We anticipate that combining standard temozolomide therapy with a pharmacological NFκB inhibitor could improve the outcome of GBM treatments in the clinic.

## Materials and Methods

### Ethics statement

All the experiments were performed according to the National Institutes of Health (NIH) guidelines and regulations. The Institutional Animal Care and Use Committee (IACUC) and Institutional Review Board (IRB) of Augusta University (animal protocol #2014–0625) approved all the experimental protocols. All animals were kept under regular barrier conditions at room temperature (21 to 25 °C) with exposure to light for 12 hours and dark for 12 hours. Food and water were offered *ad libitum*. Body weight was measured twice weekly as an indicator of overall animal health. All efforts were made to ameliorate the suffering of animals. CO_2_ with secondary method was used to euthanize animals for tissue collection at the end of the study.

### Transgenic animals

To obtain the p65^fl/fl^/LysMCre animals, p65^fl/fl^ mice^69^ were bred to B6.129P2-*Lyz2* ^*tm1(cre)Ifo*^/J (Jackson Lab) animals until homozygous animals were obtained. Genotypes were confirmed via PCR-based genotyping and p65 knockdown was confirmed via western blotting of various cell types.

### Cell lines and patient-derived xenograft (PDX)

GL261 syngeneic (C57BL/6 mouse derived) GBM cell line was obtained from Dr. Ted Johnson (Augusta University) and was authenticated in 2016. The human glioma U251 cell line was obtained from Dr. Steve Brown of Henry Ford Health System. The cell line was authenticated in July 2014 using the STR profiling method. The cell line, U251 was grown in high glucose (4.5 g/L) Dulbecco’s modified eagle’s medium (DMEM) and GL261 in RPMI (Roswell Park Memorial Institute, Thermo Scientific), supplemented with 10% fetal bovine serum (FBS), 2 mM glutamine and 100 U/ml penicillin and streptomycin at 5% CO_2_ at 37 °C in a humidified incubator. Patient-derived GBM cells (HF2303) was obtained from Dr. Tom Mikkelsen’ s lab at Henry Ford Hospital and was grown in neurosphere medium (NM), composed of DMEM/F-12 supplemented with N2 (Gibco), 0.5 mg/ml BSA (Sigma), 25 μg/ml gentamicin (Gibco), 0.5% antibiotic/antimycotic (Invitrogen), 20 ng/ml basic fibroblast growth factor, and 20 ng/ml EGF (Peprotech). Cells were maintained in culture for up to passage 10 (low passage). Patient-derived PDX GBM cells (GBM811) was obtained from North Western University and was propagated in immunocompromised NOD-SCID mice. The tumor was disintegrated into a cell suspension at the time of intracranial tumor implantation^29^.

### Animal model of GBM

Animals were anesthetized with 100 mg/kg ketamine and 15 mg/kg xylazine i.p. The surgical zone was swabbed with betadine solution, the eyes coated with Lacri-lube, and the animals were immobilized in a small animal stereotactic device (Kopf, Cayunga, CA). After draping, a 1-cm incision was made 2 mm to the right of the midline 1 mm retro-orbitally; the skull was exposed with cotton-tip applicators, and a 23 G needle tip was used to drill a hole 2 mm to the right of the bregma, taking care not to penetrate the dura. A 10 µL Hamilton syringe with a 26G-needle containing tumor cells (GL261: 10^3^, U251: 2.4 × 10^5^, and PDX GBM811: 5 × 10^4^) in a volume of 3 µl was lowered to a depth of 2.5 mm and then raised to a depth of 2 mm. During and after the injection, the careful note was made of any reflux from the injection site. After completing the injection, we waited 2–3 minutes before withdrawing in a stepwise manner. The surgical hole was sealed with bone wax. Finally, the skull was swabbed with betadine before suturing the skin.

### Chimeric mice

Human U251 and PDX GBM811 were orthotopically implanted in chimeric mice developed through our published protocol^12,34^. In brief, athymic nude mice (n = 5 for each type of chimera) were sublethally irradiated (6 Gy)^12,34^. After 24 hours, recipient mice were injected intravenously with BM cells (10–15 × 10^6^ cells) collected from donor transgenic mice (control or p65 KO mice). All mononuclear cells were separated from red blood cells using lymphocyte cell separation media (Corning, Cellgro, USA).

### *In vivo* magnetic resonance imaging (MRI)

All MRI experiments were conducted using a 7 Tesla 12 cm (clear bore) magnet interfaced to a varian console with actively shielded gradients of 49 gauss/cm and 100µs rise times or a horizontal 7 Tesla BioSpec MRI spectrometer (Brucker Instruments, Bellerica, MA) equipped with a 12 cm self-shielded gradient set (45 gauss/cm max). Detailed MRI procedures were adopted from our several previous publications^12,29,34^. An appropriate state of anesthesia was obtained with isoflurane (2.5% for induction, 0.7% to 1.5% for maintenance in a 2:1 mixture of N_2_: O_2_). After positioning using a triplanar FLASH sequence, MR studies were performed using pre-contrast T1, T2-weighted and post-contrast T1-weighted MRI scans with the following parameters: (1) Standard T1-weighted multislice sequence (TR/TE = 500/10 ms, 256 × 256 matrix, 13–15 slices, 1 mm thick slice, 32 mm field of view (FOV), # of averages = 4); (2) T2-mapping sequence (2D multi-slice, multi-echo (MSME) sequence, TE = 10, 20, 30, 40, 50, 60 msec, TR = 3000 msec, 256 × 256 matrix, 13–15 slices, 1 mm thick slice, 32 mm field of view (FOV), # of averages = 2). Post contrast T1WI was used to determine the volume of tumors in vehicle and p65 deleted mice by drawing irregular ROI to encircle the whole tumor in each image section-containing tumor using ImageJ software, and the area was then multiplied by the thickness of image slice to determine the volume (cm^3^). Two investigators blinded to the animal groups determined tumor volume.

### Characterization of immune cell and CSC populations

Freshly isolated tumor, spleen and BM samples from each group were passed through a 40µm-cell strainer to make single cells. Cells were labeled with antibodies (Bio Legend) against immune cell antigens such as CD45, CD4, CD8, Gr1, CD11b, F4/80, CD68, CD206 (mannose receptor, M2), and CD86 (mature dendritic cells, M1) in the tumor as well as in spleen and BM. CSC antigens such as CD133 and CD44 were analyzed on tumor cells (CD45−). Immunogenicity or presence of MHC1 (H2Ld-H2Db) on GL261 tumor cell line was analyzed using specific antibody (BioLegend). Flow cytometry data were acquired using Accuri C6 machine (BD Biosciences) and analyzed by BD Accuri C6 software.

### Histology

After MRI at day 22, control mice (n = 3) and p65 KO mice (n = 3) were euthanized and brains were collected for paraffin fixed tissue sections and later stained to determine histology through standard H&E, expression of CD11b (Abcam) and p65 (Cell Signaling Technology) through immunofluorescence, expression of proliferation marker Ki67 (Dako) and apoptosis marker cleaved caspase 3 (Cell Signaling Technology) through immunohistochemistry using standard methods. Mice staining data were validated using human GBM tumors (n = 10) collected from Augusta University Biorepository under an IRB approved protocol. The data were acquired using an automated all-in-one microscope (BZ-X710, Keyence).

### Cell sorting, protein array, and functional assay

CD11b positive myeloid cells from tumors from p65 control and p65KO mice were harvested using positive selection method (Miltenyi Biotec). Purity for each population ranged from 90–99%, as detected by flow cytometry. Sorted CD11b cells were used for total protein isolation and were processed for customized mouse cytokine array (44 factors) (Ray Biotech). Membranes were imaged using Las-3000 imaging machine (Fuji Film, Japan). All signals (expression intensity) emitted from the membrane were normalized to the positive control spots of the corresponding membrane using ImageJ software. A web-based Ingenuity Pathway Analysis (IPA) interfaced for the pathway and network analysis (www.qiagenbioinformatics.com)^12^. Freshly isolated total CD3+T cells isolated from normal spleen and lymph nodes were labeled with CFSE dye per the manufacturer’s protocol (Life Technologies, NY), and were stimulated with 10 μg/mL plate-bound anti-CD3, 2.5 μg/mL soluble anti-CD28, and cocultured with CD11b+ cells. Non-stimulated T cells and CD11b monoculture were used as a control for the assay. T cell proliferation was monitored 72 hours later by flow cytometry^70^. To identify the direct effect of proinflammatory cytokines such as IFN-γ, TNF-α, and IL1-β on T cell proliferation, sorted T cells were cultured similarly. Stimulated T cells were treated with IFN-γ (100 U/ml), TNF-α (100 ng/ml), or IL1-β (100 ng/ml), or a combination of the three. Following co-culture, T cells were labeled with CD4 and CD8, and CD11b cells were labeled with CD86 mature DC marker to compare the cell proliferation at all the conditions. Co-culture supernatant was tested for cytokine expression using membrane-based protein array, as described above.

### Western blot analysis

Bone marrow cells from p65 KO mice (n = 2) were collected through the standard protocol. CD11b positive myeloid cells and CD11b negative cells were harvested using positive selection method (Miltenyi Biotech). Brain tissue from P2 pups was dissociated using the Neuronal Dissociation Kit (Miltenyi #130–092–628), and microglia were isolated using CD11b positive selection (Miltenyi #130-093-636). Cells and tissues were processed for protein isolation using Pierce RIPA buffer (Thermo Scientific, USA). Protein concentrations were estimated with Pierce, BCA protein assay kit (Thermo Scientific, USA), and separated by standard Tris/Glycine/SDS gel electrophoresis. Membranes were incubated with primary antibodies against p65 (1:1000, Cell Signaling), and β-actin (1:3000, Sigma) followed by horseradish peroxidase-conjugated secondary antibody (1:5000, Bio-Rad). The blots were developed using a Pierce Super Signal West Pico Chemiluminescent Substrate kit (Thermo Scientific, USA). Western blot images were acquired by Las-3000 imaging machine (Fuji Film, Japan).

### Sphere formation assay

Assay was performed using a standard protocol. Briefly, a total of 1 × 10^4^ GL261 or HF2303 (patient GBM derived cells) tumor cells were plated on matrigel in 96 wells plate. The tumor cells were cultured with the 50% conditioned medium derived from the culture of wild-type BMDMs, control BMDMs or p65 KO BMDMs. Images were acquired right after adding conditioned medium (day 0), on day 2 and day 5 of culture with 5x magnification using Amscope inverted microscope equipped with a digital camera (Amscope).

### Human data analysis

The mRNA expression data from brain cancer patient’s datasets were analyzed using Oncomine data (TCGA Brain, and Albino Brain) (www.oncomine.org) and GEO dataset (GSE4630)^35^.

### Statistical analysis

Quantitative data were expressed as mean ± SD and analyzed through analysis of variance (ANOVA) or Student t-test. Differences were considered statistically significant at p-value < 0.05.

## Electronic supplementary material

Supplementary information
