# Supplementary material for: Canonical NFκB signaling in myeloid cells is required for the glioblastoma growth
Source: Sci Rep. 2017 Oct 23;7:13754. doi: 10.1038/s41598-017-14079-4 (PMC5653749; doi:10.1038/s41598-017-14079-4)
Supplement: Supplementary file 1 — Supplementary information [file 41598_2017_14079_MOESM1_ESM.doc]

**TITLE:** Canonical NFκB signaling in myeloid cells is required for the glioblastoma growth.

**Authors:** B.R. Achyut^1#^, Kartik Angara^1^, Meenu Jain^1^, Thaiz Borin^1^, Mohammad Rashid^1^, ASM Iskander^1^, Roxan Ara^1^, Ravindra Kolhe^2^, Shelby Howard^4^, Natasha Venugopal^4^, Paulo C. Rodriguez^3^, Jennifer W. Bradford^3,4#^, Ali S. Arbab^1#^

**SUPPLEMENTARY FIGURES AND LEGENDS**

**Supplementary Figure 1**. Hematoxylin and Eosin stain showing decreased GBM growth in p65 KO mice compared to controls. Shown is one of the representative images from each group.


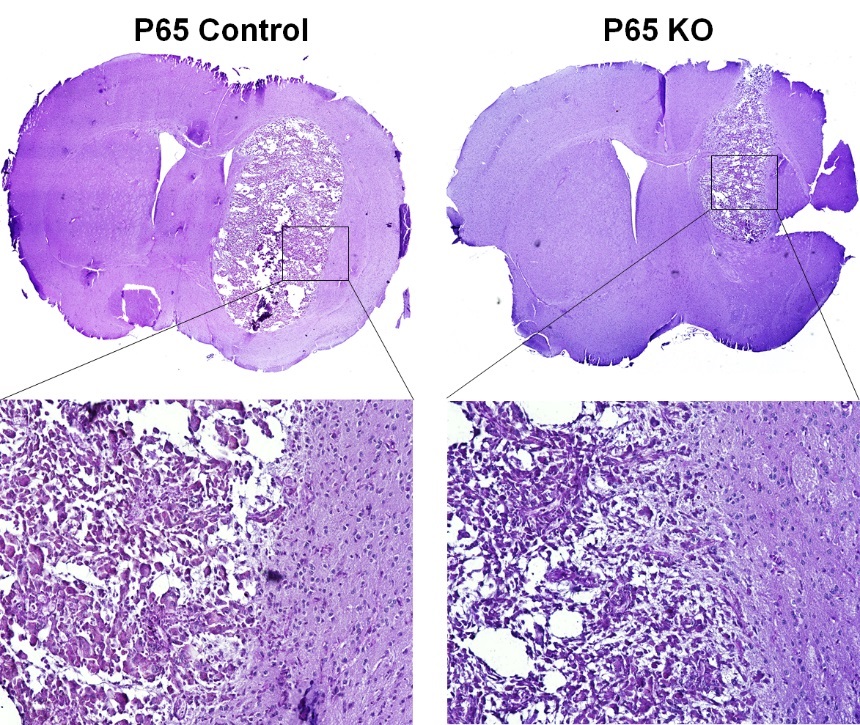


**Supplementary Figure 2**. The proliferative and anti-apoptotic role of myeloid NF-κB signaling in GL261 TME: Paraffin-embedded tumor sections from p65 control and p65 KO mice were stained with (A) proliferation marker Ki67 and (B) apoptosis marker cleaved caspase 3. p65 KO tumors showed decreased Ki67 and increased cleaved-caspase 3 compared to p65 control tumors. Shown is one of the representative images from each group. Quantitative data are expressed in mean ±SD. ** P<0.01 and ***P<0.001.

**
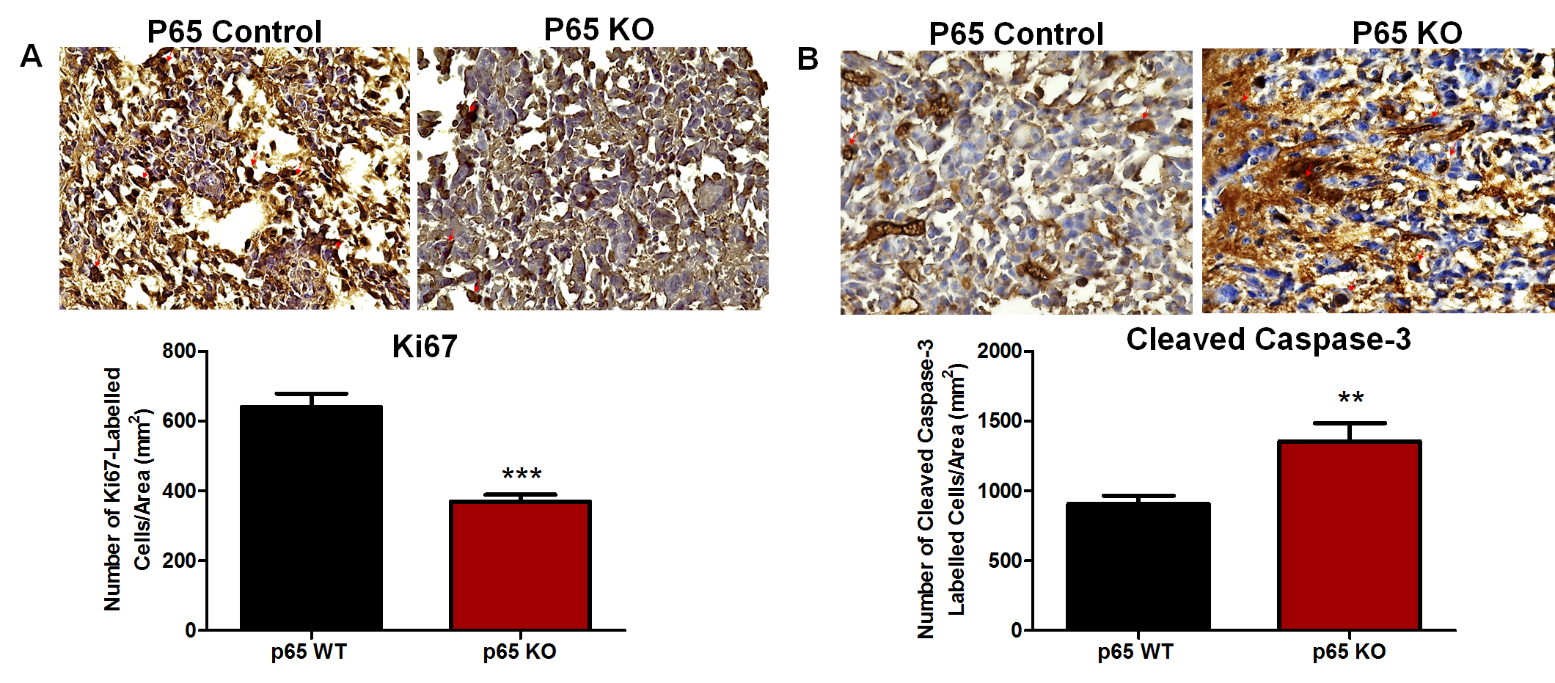
**

**Supplementary Figure 3**. Western blot data showing uncropped images presented in Figure 1. Left panel showing p65 (top band) and β-actin (bottom band) for Figure 1C. Right panel showing p65 (top band) and β-actin (bottom band) for Figure 1D. Please note that left two lanes were cropped for Figure 1D, which represent positive control lanes for p65 expression. L929 cells treated with TNF-α (100 ng/ml) and without treated were added as a control group.

**
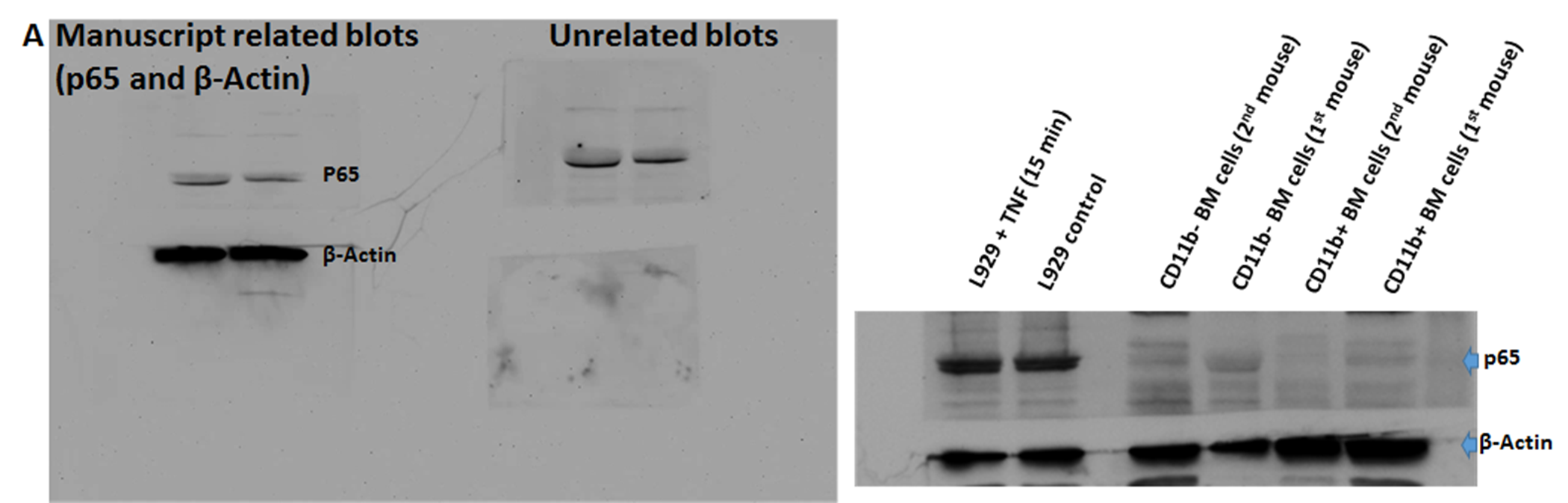
**

**Supplementary Figure 4**. Flow data showing no changes in (A) CD45+CD4+ T cells and (B) CD45-CD133+ hematopoietic stem cells in the p65 control and p65 KO GBM microenvironment. (C) The MHC1 expression on GL261 murine glioblastoma cell line. MHC1 marker (H2Ld-H2Db) expression on GL261 cells was analyzed using flow cytometry. Shown is one of the representative images from each group. Quantitative data are expressed in mean ±SD.

**
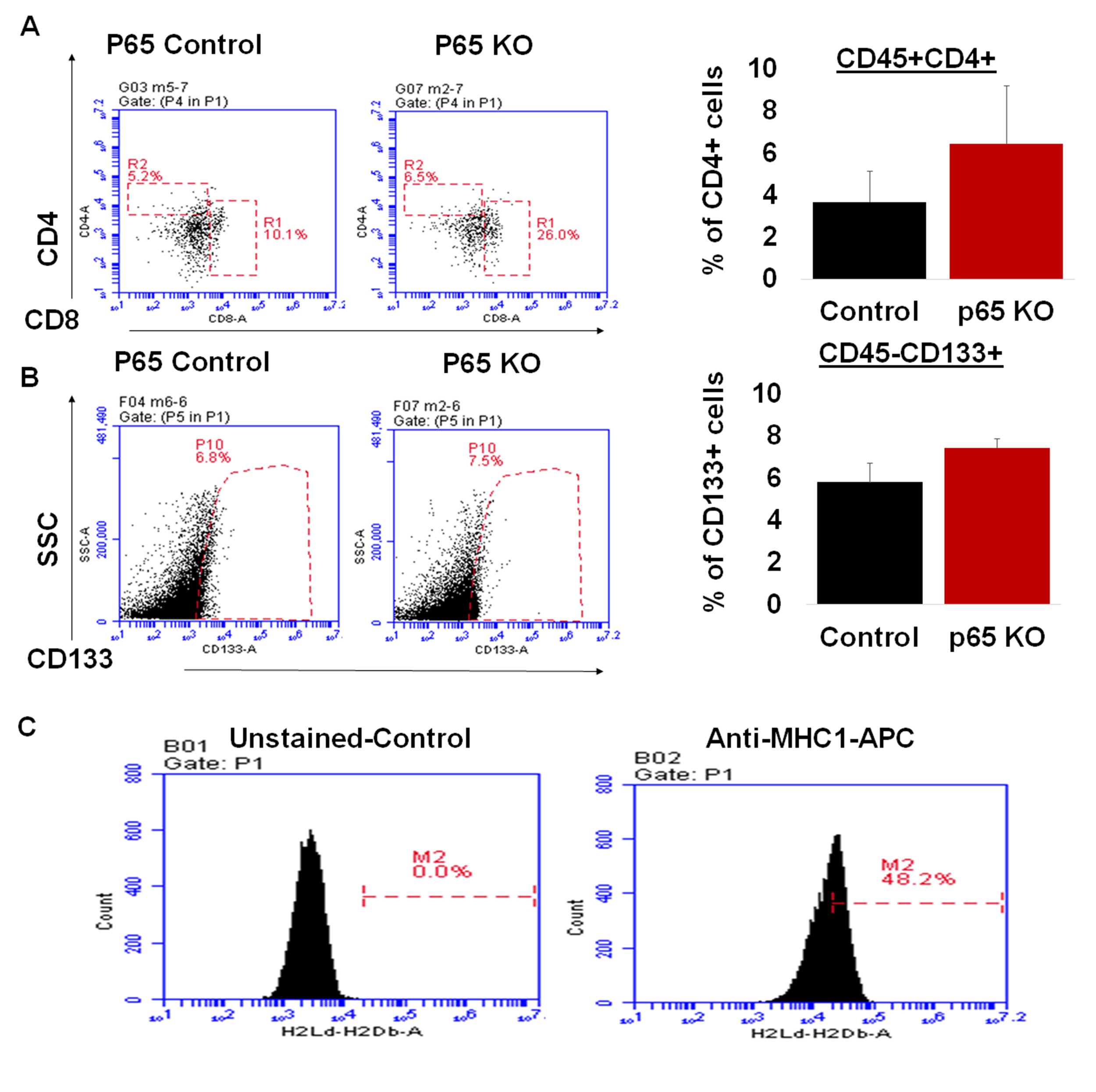
**


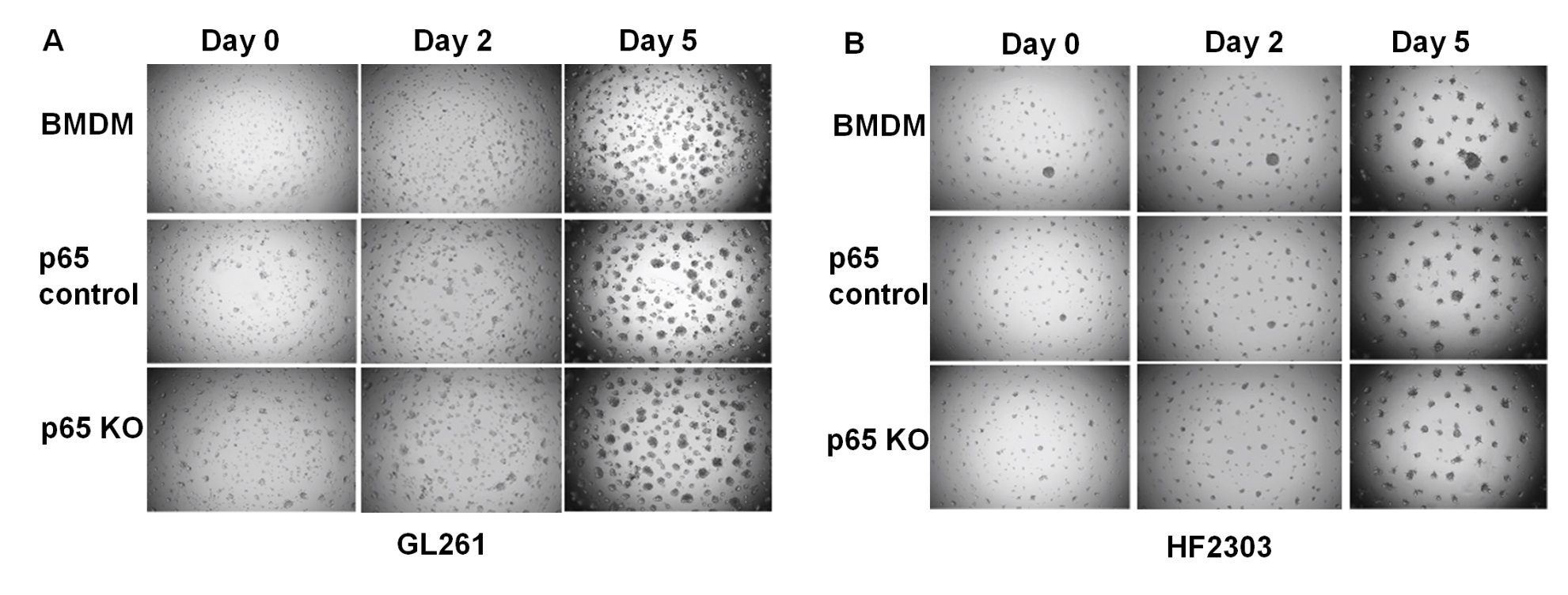
**Supplementary Figure 5.** Tumor cell (GL261 and HF2303) spheroid formation assays were performed in the presence of 50% conditioned medium derived from 24 hours’ culture of wild-type, control or p65 KO BMDMs. No difference in spheroid size was observed in the culture of (A) GL261 or (B) HF2303 tumor cells. Shown is one of the two experiments performed.

**Supplementary Figure 6**. Membrane-based cytokine array data involving p65 KO and p65 control tumors were annotated with their gene symbol. Data were analyzed through the use of Ingenuity Pathways Analysis (IPA) using gene symbols and their intensity fold change values. The web-based pathway analysis discovered that the significantly altered factors predominantly belonged to key molecular pathways such as (A) HMGB1 mediated control of E-selectin (SELE), TNF-α, ICAM1, and MCP1. (B) NF-κB mediated control of IL-1, TNF-α, and EGF. (C) TREM1 signaling mediated control of TNF-α, IL-6, MCP-1, and MIP-1α. These copyright images were included after receiving the written permission from QIAGEN Silicon Valley.


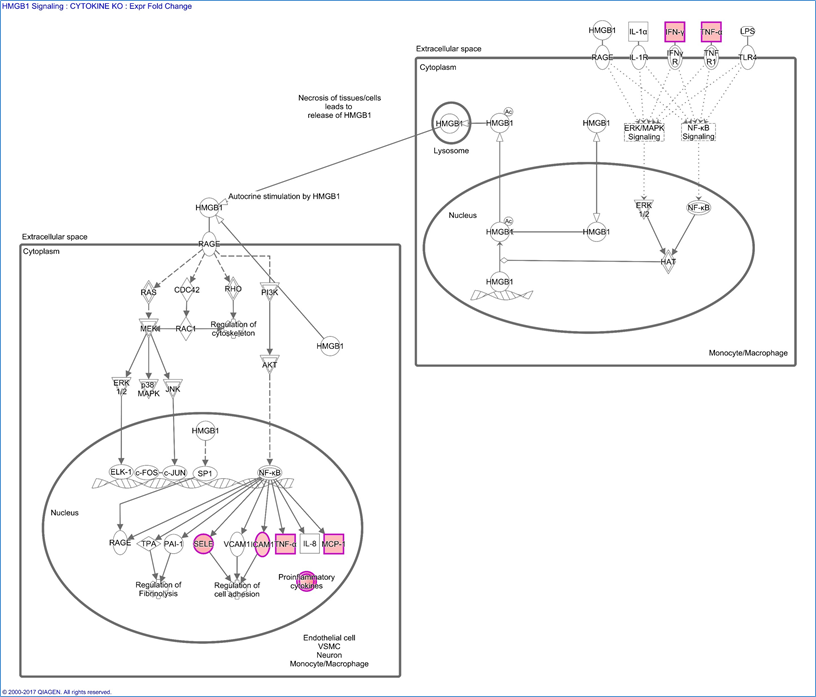


**HMGB1**

**A**

**B**


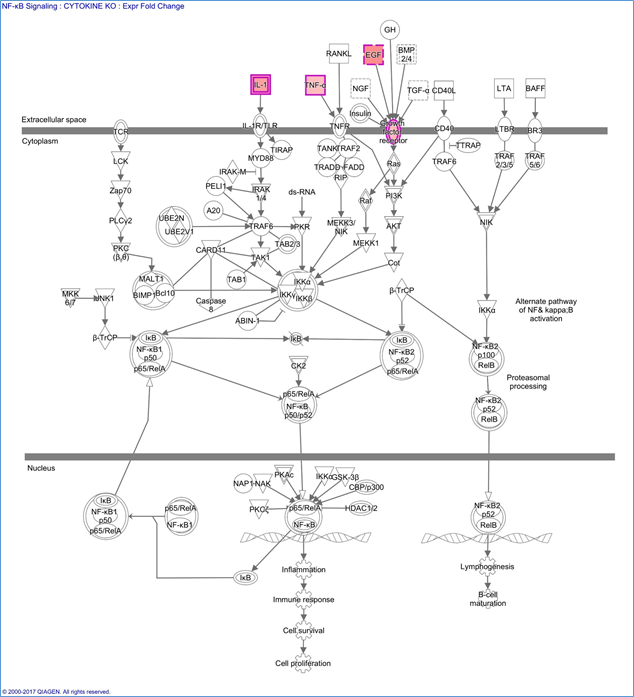


**NFκB**

**C**


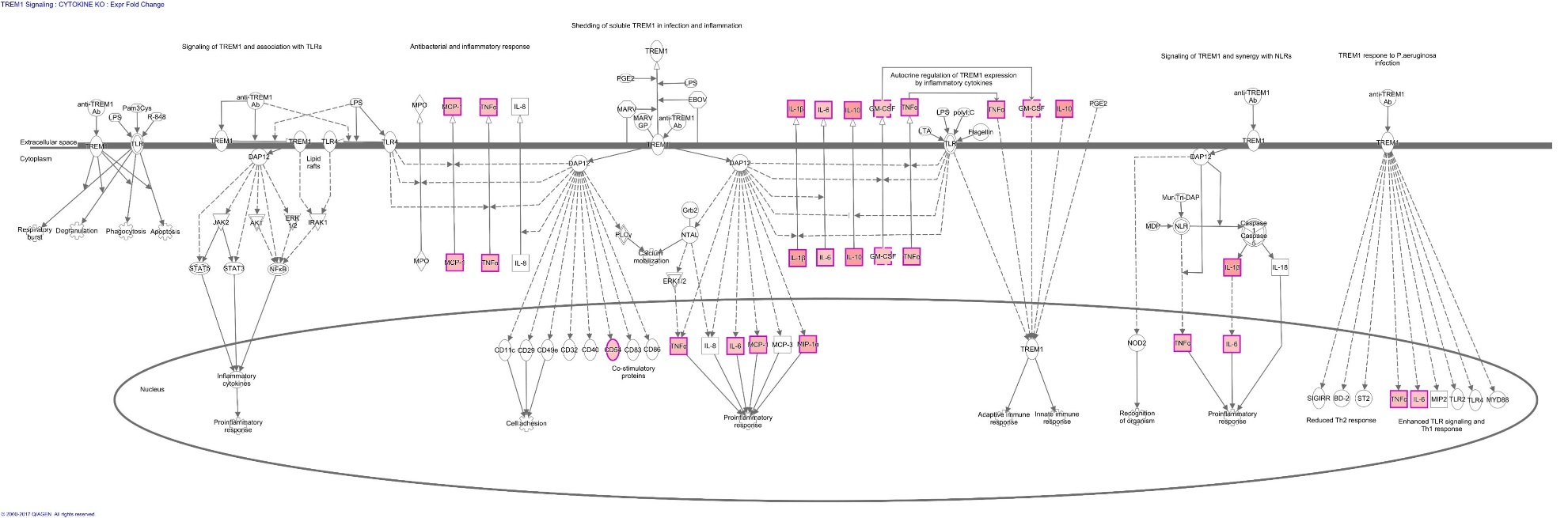


**TREM1**


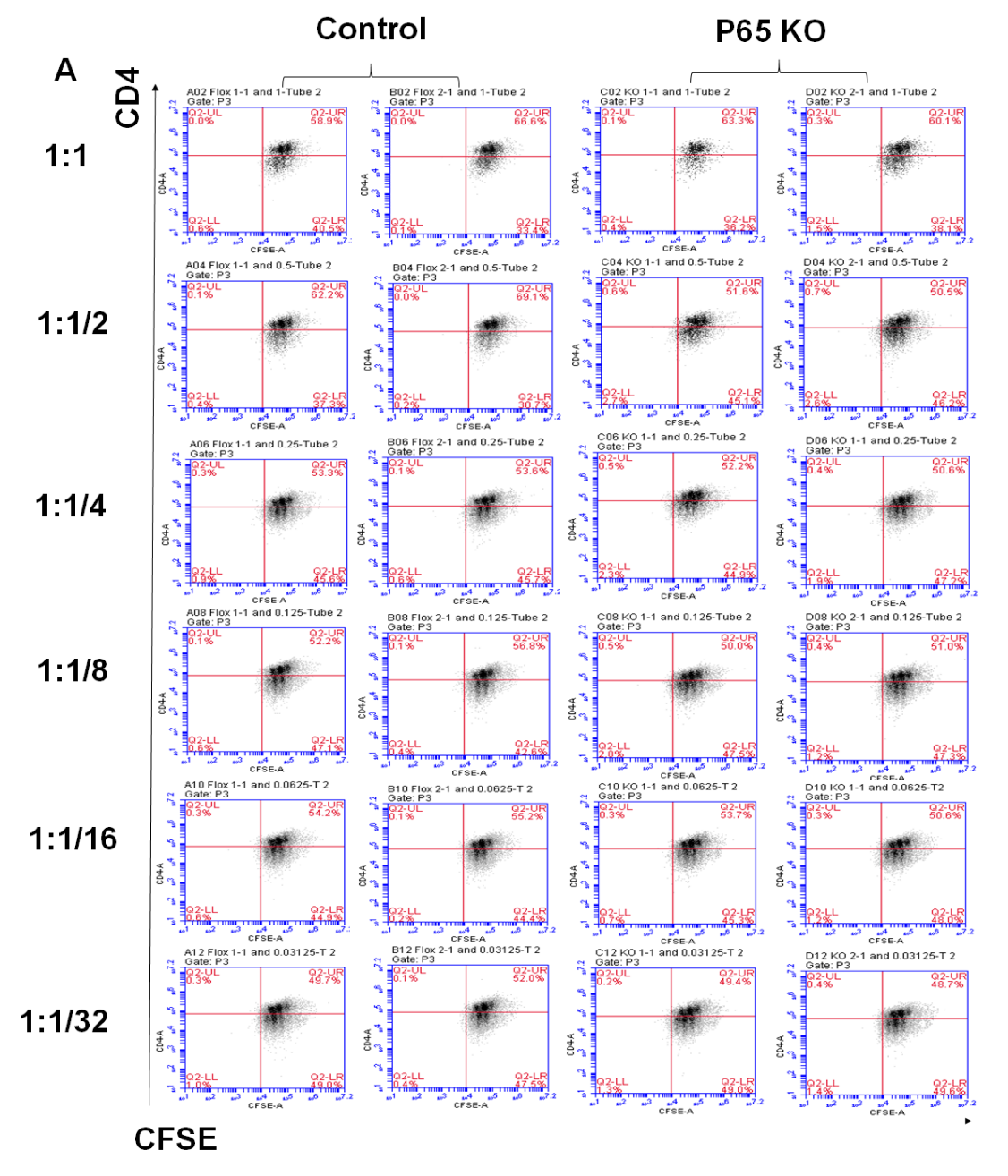
**Supplementary Figure 7.** Flow data showing (A) CD4 proliferation was decreased at the 1:1/2 co-culture of normal T cells and p65KO tumor sorted CD11b+ cells compared to p65 control CD11b+ cells. (B) CD8 proliferation was increased at the 1:1/2, 1:1/8 and 1:1/32 co-cultures in the p65KO group compared to p65 control group, indicating the anti-proliferative role of myeloid canonical NF-κB signaling on CD8+T cells under tumor condition.

**
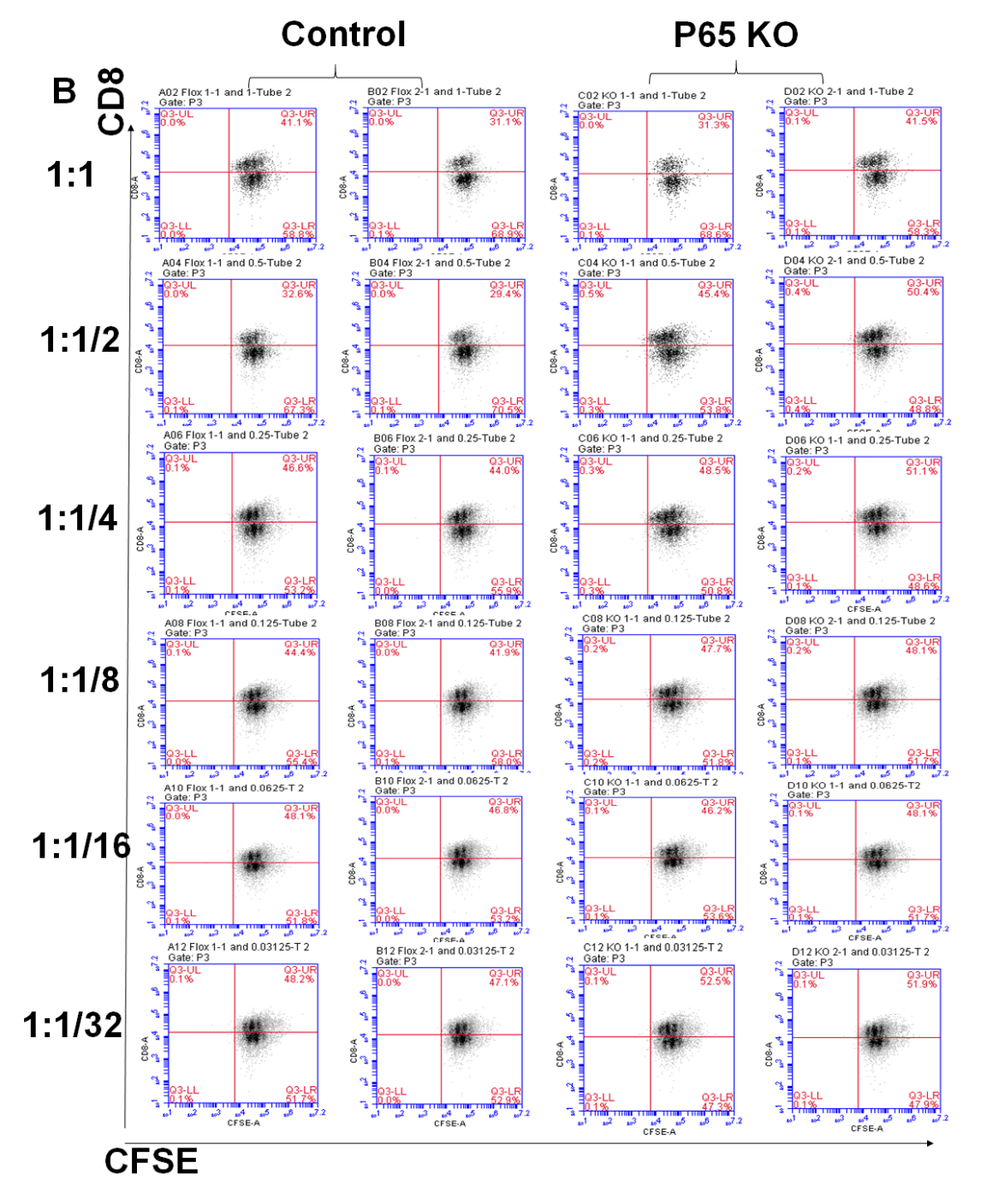
**

**Supplementary Figure 8.** **Treatment of proinflammatory Th1 cytokine increases CD8+ T cell proliferation in culture.** Control group displayed high proliferation (93.2%, B) when stimulated with the anti-CD3 and anti-CD28 compared to non-stimulated group (A). We found that treatment of proinflammatory cytokines IFN-γ, TNF-α, IL1-β alone or in combination (C, D, E, and F) did not increase total T cell proliferation. In fact, TNF-α, IL1-β or combination of all three cytokines showed a trend of decreased T cell proliferation (89.4%-84%, D, E, and F) compared to stimulated control group. Interestingly, cytokine treatment alone or combination of all decreased CD4+ T cells (R1 gate) and increased CD8+ T cells (R2 gate), when specific T cell populations were compared for the proliferation of control groups.


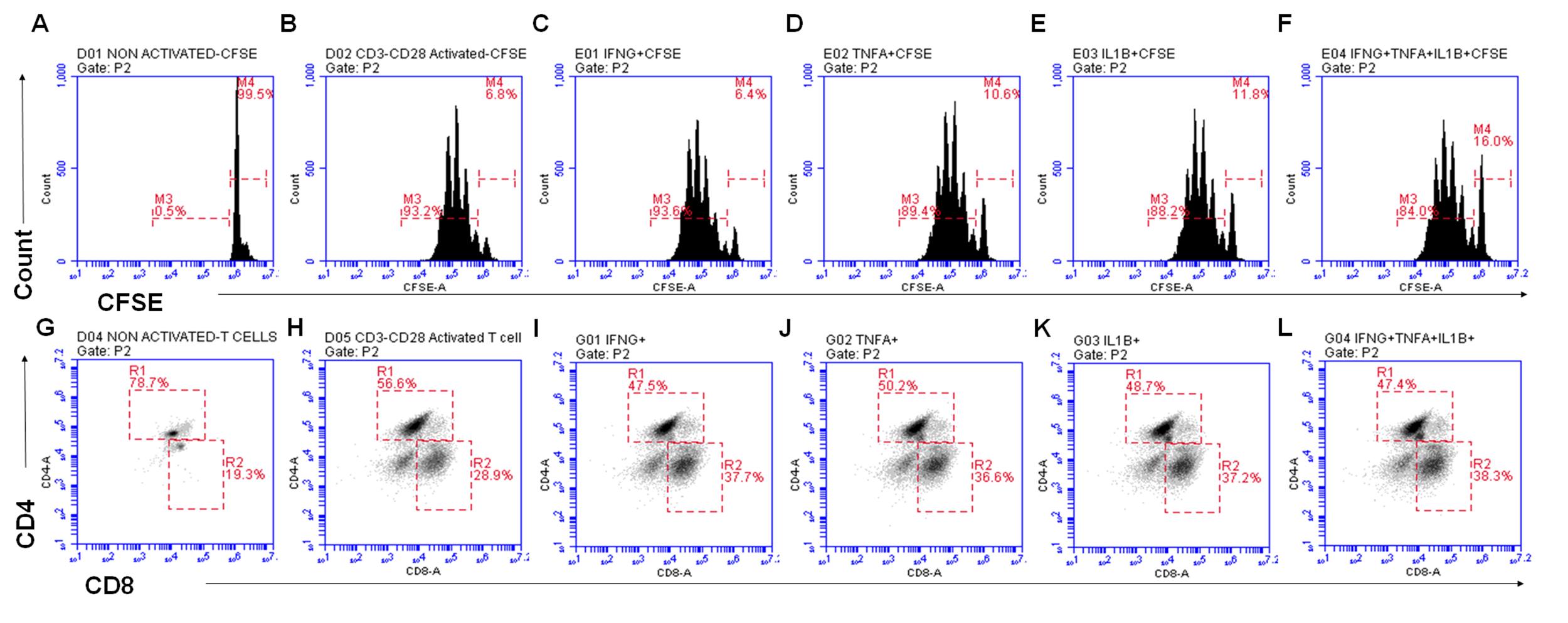


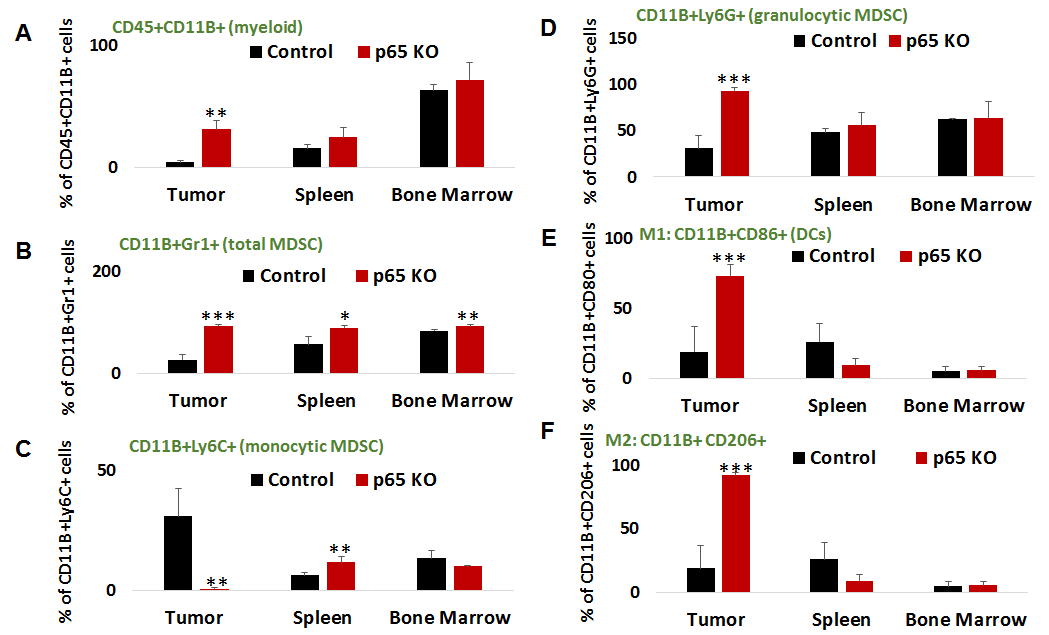
**Supplementary Figure 9**. Flow cytometric analysis of human GBM TME (GBM811) identified that p65KO chimera had slightly increased total CD45+ leukocytes (data not shown), (A) increased total CD11b+, increased F4/80+ macrophages (data not shown), increased CD68+ macrophages (data not shown), (B) increased Gr1+CD11b+ (total MDSCs), (C) decreased Ly6C+CD11b+ (monocytic MDSCs), (D) increased Ly6G+CD11b+ (granulocytic MDSCs), (E) increased CD11b+CD86+ (mature DCs or M1 macrophages), and (F) increased CD11b+CD206+ (M2 macrophages) compared to TME in control chimera. Shown is one of the two experiments performed. Quantitative data is expressed in mean ±SD. ** P<0.01 and ***P<0.001.
